# Investigation of the Effects of Molecular Parameters on the Hemostatic Properties of Chitosan

**DOI:** 10.3390/molecules23123147

**Published:** 2018-11-30

**Authors:** Zhang Hu, Sitong Lu, Yu Cheng, Songzhi Kong, Sidong Li, Chengpeng Li, Lei Yang

**Affiliations:** Faculty of Chemistry and Environmental Science, Guangdong Ocean University, Zhanjiang 524088, China; smelilst@163.com (S.L.); Vickygirl7442@163.com (Y.C.); kongsongzhi@126.com (S.K.); sidongligdou@163.com (S.L.); lcp0802@126.com (C.L.)

**Keywords:** chitosan, hemostatic properties, molecular parameters, effects

## Abstract

Hemorrhea is one of the major problems in war, trauma care, and surgical operation that threaten the life of the injured and patients. As a novel polymeric hemostatic agent, biodegradable chitosan can stop bleeding through a variety of approaches. In this paper, chitosan with various molecular parameters was prepared from chitin as raw material through deacetylation, oxidative degradation, hydrophilic modification, and salt formation reactions. The influence of different polymer parameters on the hemostatic effects of chitosan was investigated by in vitro coagulation time and dynamic coagulation assay. The results showed that when the molecular weights were high (10^5^–10^6^) and approximate, the coagulation effect of chitosan improved with a decrease of the deacetylation degree and achieved a prominent level in a moderate degree of deacetylation (68.36%). With the same degree of deacetylation, the higher the molecular weight of chitosan, the better the procoagulant effect. The substituent derivatives and acid salts of chitosan showed significant procoagulant effects, especially the acid salts of chitosan. In addition, the hemostasis mechanism of chitosan with various parameters was preliminarily explored by analyzing the plasma recalcification time (PRT). The efforts in this paper laid a basis for further study of the structure–activity relationship and the mechanism of chitosan hemostasis.

## 1. Introduction

In the battlefield, large-scale disasters, and other accidental wounds, uncontrolled hemorrhage leads to over 30% of trauma deaths worldwide and more than half of those occur before emergency care can be reached [1,2]. Surgery is one of the methods to control bleeding, but it is not timely and effective to control bleeding only by surgical operation. Therefore, more and more attention has been paid to the development of alternative methods of rapidly stopping bleeding, mainly including various forms of novel effective hemostatic agents such as sponges [3,4], powders [5,6], hydrogels [7,8], films [9,10], and fibers [11].

Among numerous hemostatic materials, chitosan is a linear amino-polysaccharide material with great potentiality for development. It is obtained by deacetylation of chitin, which is the second most natural polymer after cellulose on earth. Chitin is commonly found in crustacean shells, insect cuticles, and many fungi, and is currently industrially extracted from marine shell waste streams [12,13,14,15,16]. The chemical structure of chitosan consisting of 2-amino-2-deoxy-d-glucose and 2-acetamido-2-deoxy-d-glucose units linked by β-1, 4 glycosidic bonds is similar to that of mucopolysaccharide [17,18,19,20]. Due to its biocompatibility, biodegradability, non-toxicity, antimicrobial, and hemostatic properties, chitosan has been considered as an attractive candidate for hemostasis in clinical applications [21,22]. Currently, some commercial hemostatic agents have been developed using chitosan as the main component and approved by the Food and Drug Administration of the United States (FDA), such as Celox powder and HemCon bandages. However, it is clear that many challenges still exist in extensively practical applications. Recently, more and more attention has been paid to chitosan (CS)-based hemostatic materials which have been shown to possess excellent properties, such as no cytotoxicity, no hemolysis, and good biological compatibility [23]. Nevertheless, the hemostatic effects of these CS-based materials still need further improvement.

Generally, the structure–activity relationships of natural products are very subtle. The biological functions may be dramatically altered by subtle changes in the molecular structure. Most studies have shown that the biological activity of chitosan is a function of several structural factors, such as their degree of *N*-acetylation and molecular weight [24,25,26]. However, at present very few studies have reported on the relationship of the structure of chitosan and its hemostatic activity. In order to translate scientific investigations into clinical applications, a systematic approach is needed, in which the factors affecting the study outcomes should be considered in detail. Therefore, this paper tried to explore the influence of the structural parameters of chitosan, including degree of deacetylation (DD), molecular weight (MW), substituent group (SG), and salt molecule on its procoagulant effects, so as to provide a theoretical basis for developing highly effective chitosan-based hemostatic materials.

## 2. Results and Discussion

### 2.1. Preparation of Chitosan with Different Molecular Parameters

**Figure 1:**
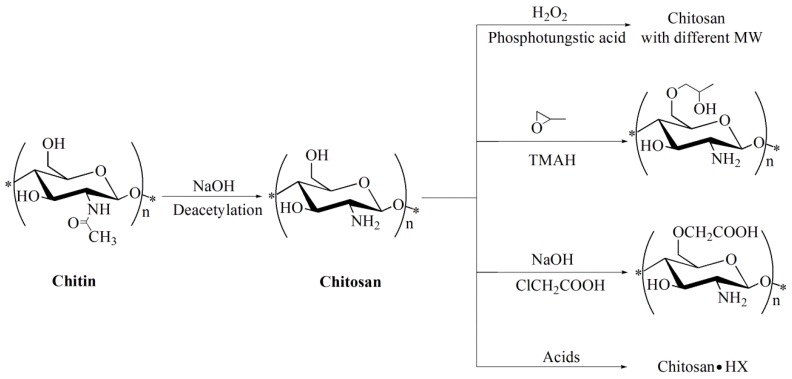
Preparation of chitosan with different molecular parameters.

The main preparation processes of chitosan with different molecular parameters were illustrated in Figure 1. Chitosan with different degrees of deacetylation, ranging from 52.68% to 92.21%, was prepared by deacetylation of chitin as the starting material treated under strong alkali, which was marked as CS-D1, CS-D2, CS-D3, and CS-D4, respectively, as shown in Table 1. Through oxidative degradation of CS-D3 as basic material, a series of chitosan with molecular weights ranging from 485 to 25 kDa was produced and marked as CS-M1, CS-M2, CS-M3, and CS-M4, respectively. *O*-carboxymethyl chitosan was prepared by carboxymethylation with a degree of substitution (DS) of 63.13% and marked as CS-S1. Through alkalization pretreatment, *O*-hydroxypropyl chitosan was obtained by the reaction of chitosan with propylene oxide in the presence of tetramethylammonium hydroxide (TMAH) with a DS of 58.36% and marked as CS-S2. Through chemical reactions with acids, chitosan hydriodide, chitosan acetate, chitosan lactate, and chitosan gentisate were obtained and marked as CS-A1, CS-A2, CS-A3, and CS-A4, respectively.

### 2.2. FTIR Analysis

**Figure 2:**
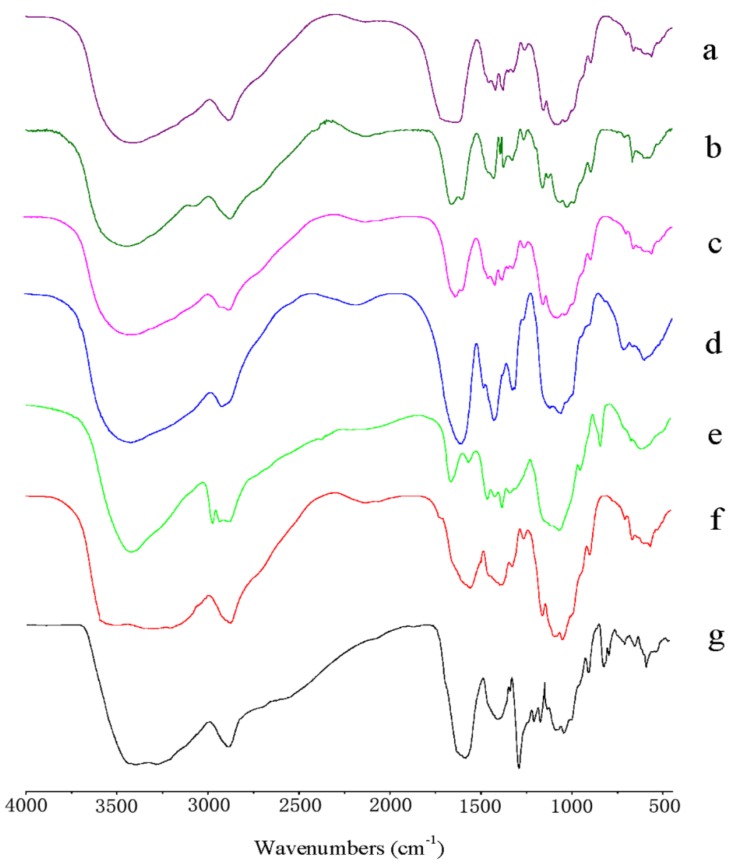
FTIR spectra of: (a) chitin; (b) CS-D3; (c) CS-M1; (d) CS-S1; (e) CS-S2; (f) CS-A2; (g) CS-A3.

FTIR spectroscopy is sensitive to the chemical structures of molecules and suitable for the determination the activated groups of chemical components. The FTIR spectra of chitosan with varied molecular parameters are shown in Figure 2. From Figure 2a, chitin showed a broad absorption band at 3100–3500 cm^−1^, corresponding to -OH stretching vibrations, which overlapped the -NH stretching vibrations. The band found at 1650 cm^−1^ was attributed to an amide I (C=O), and the amide II band (around 1550 cm^−1^) of a secondary amide was weak and almost completely covered by the amide I band. The amide III band resulting from C–N stretching appeared at 1374 cm^−1^. The absorption band at 1040 cm^−1^ corresponded to C–O stretching vibrations. After deacetylation of chitin, chitosan (CS-D3 & CS-M1) showed the relatively weak characteristic absorption peaks of amide I (C=O) at about 1650 cm^−1^, and the band at 1600 cm^−1^ related to the bending vibration of the N-H bonds of primary amines was obvious, which were consistent with previous reports [27], as shown in Figure 2b,c. Compared with chitosan (CS-D3 & CS-M1), *O*-carboxymethyl chitosan (CS-S1) showed the appearance of new absorptions at 1593 and 1411 cm^−1^, which were ascribed to the asymmetric and symmetric stretching vibrations of -COO, as shown in Figure 2d, indicating the carboxymethylation of chitosan. Similarly, in the spectrum of hydroxypropyl chitosan (CS-S2) given in Figure 2e, new absorption bands at 2973 and 1376 cm^−1^ were assigned to the stretching and bending of the -CH_3_, which were in agreement with the results in References [28,29], confirming the successful hydroxypropylation of chitosan. In the spectrum of chitosan acid salts, as shown in Figure 2f,g, compared with that of the original chitosan (CS-D3), the broad absorption band at about 3400 cm^−1^ became wider and weaker, which might be due to the formation of NH_3_^+^ in the molecule. Meanwhile, the characteristic absorption band of the amine group at about 1602 cm^−1^ disappeared, showing that the amine group was protonated. Additionally, the peaks at about 1565 and 1400 cm^−1^ corresponding to the asymmetric and symmetric stretching vibrations of the carboxylate, were observed. Consequently, the ionic reaction between chitosan and acids was successfully performed. In conclusion, all the evidence from the FTIR spectra indicated that chitosan with varied molecular parameters had been successfully prepared.

### 2.3. Effect of DD on the Procoagulant Activity of Chitosan

**Figure 3:**
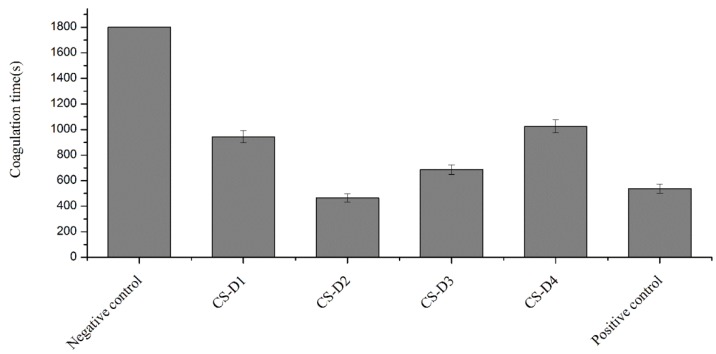
Effect of DD of chitosan on coagulation time.

The DD of chitosan indicates the content of the free amino group in the chitosan molecular chain, which is closely related with the dissolvability, viscosity, ion exchange capacity, flocculation performance, and properties of chemical reaction with the amino group. Therefore, DD is an important indicator of the quality of chitosan. The hemostatic properties of chitosan derive in part from its net positive charge, which initiates aggregation of negatively charged red blood cells. The effects of chitosan with high and approximate MW (10^5^–10^6^) and different DD on the coagulation time of rabbit blood in vitro were investigated. The results are illustrated in Figure 3. The anticoagulant rabbit blood in the negative control group did not coagulate at all within 30 min. Compared with the negative control group, chitosan powders with different DD had significant procoagulant effects in vitro, with average coagulation time ranging from 563 s to 1025 s. Compared with the positive control group, only CS-D2 (with DD 68.36%) had a similar coagulation time (*p* < 0.05). The coagulation time of other chitosan groups was significantly higher than that of the positive control group (537 s). It was observed that CS-D2 with a moderate DD had the most significant procoagulant effect, while CS-D4 with a high DD and CS-D1 with a low DD had relatively poor procoagulant effects. These results were in agreement with previous reports [30]. Studies have shown that the ability of chitosan to initiate coagulation was related to the percent of deacetylation and was more dependent on the number of protonated amine groups [31,32]. Besides, considered from the molecular structure, an appropriate quantity of amino groups in the molecules of chitosan may enable chitosan to construct a mesh-like spatial structure, which promoted interaction between blood components and chitosan and facilitated formation of strong blood clotting. In contrast, chitosan with a higher degree of deacetylation had more amino groups and hydroxyl groups in the molecules, which were more likely to form stronger hydrogen bonds inside the molecules, leading to a unique crystalline structure of chitosan that could hardly interact with blood components to promote coagulation.

### 2.4. Effect of MW on the Procoagulant Activity of Chitosan

**Figure 4:**
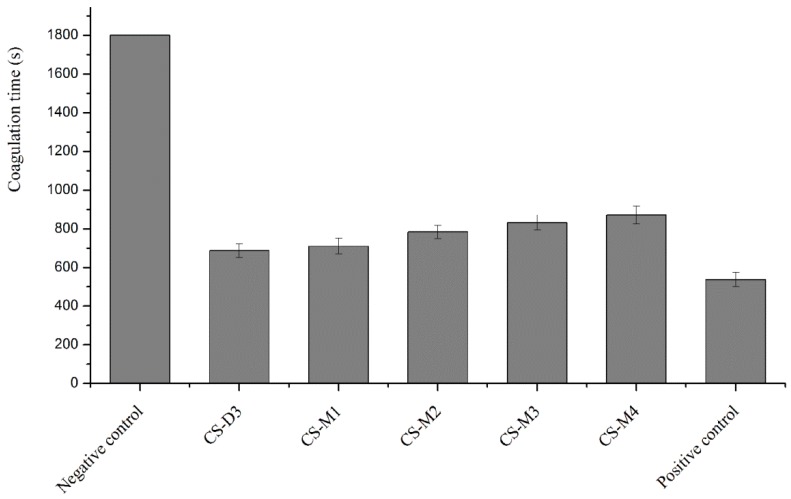
Effect of MW of chitosan on coagulation time.

MW is another important structural parameter of chitosan and has significant effects on its chemical and biological properties [33,34]. Chitosan with different MW differs significantly in its properties, some of which even presents only with a specific MW [35]. The effect of chitosan with similar DD and different MW on coagulation time of rabbit blood was investigated in vitro and the results are illustrated in Figure 4. It was observed that chitosan with different MW had certain procoagulant effects in vitro, with coagulation times of 686 s, 709 s, 783 s, 831 s, and 871 s, respectively, showing significant difference as compared with the negative control group. A general trend can be seen; that the greater MW of chitosan with similar DD, the better the coagulation effect. Chitosan facilitated hemostasis possibly by interacting with erythrocytes and linking them together to establish a cellular clot or hemostatic plug. It was due to the increase in entanglement between polyelectrolyte molecules, which was closely related to the relative MW and the polymeric structure of chitosan [36,37].

### 2.5. Effect of SG on the Procoagulant Activity of Chitosan

**Figure 5:**
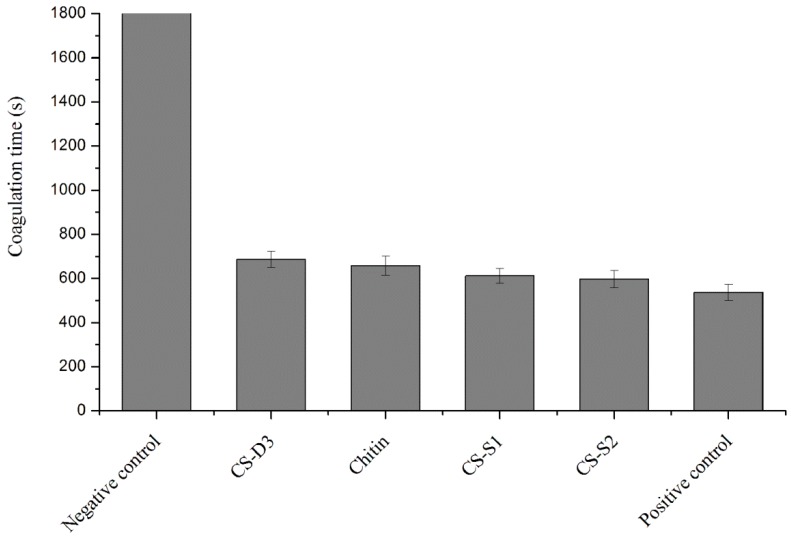
Effect of substitution group of chitosan on coagulation time.

Chitosan possesses a large number of amino and hydroxyl groups that permit it to be easily modified by chemical processes such as acylation, *N*-phthaloylation, alkylation, Schiff base formation, reductive alkylation, tosylation, *O*-carboxymethylation, *N*-carboxyalkylation, and graft copolymerization [38]. Although there were numerous studies on chitosan-based composite materials as surgical hemostatic agents, to the best of our knowledge, there were limited numbers of studies on the hemostatic capacity of single chitosan substituted compounds. The effect on blood coagulation time of chitosan with different SGs was investigated in vitro, as shown in Figure 5. The investigation results revealed that chitosan and its substituted derivatives had significant in vitro procoagulant effect when compared with the negative control group. Relative to chitosan (CS-D3), water-soluble substitution (CS-S1 and CS-S2) enhanced the procoagulant effect. It was probably because water-soluble substitution improved the protonation ability of amino groups in the molecular chain of chitosan. Although the hemostatic mechanism of chitosan has not been clearly defined, many studies demonstrated that the procoagulant performance of chitosan involved the agglutination of red blood cells (RBCs). The more positively charged the amino group on CS, which attracted the negatively-charged RBCs to agglutinate, the more easily clotting is promoted. Compared with chitosan (CS-D3), chitin with 90% acetyl substituent had a higher procoagulant effect due to its strong platelet attraction to promote coagulation [39]. Studies showed that chitin might retain platelets to form a structure similar to a platelet/fibrin mesh structure that can promote blood coagulation, which was related to its unique β-crystalline structure [40].

### 2.6. Effect of Chitosan Acid Salts on the Procoagulant Activity of Chitosan

**Figure 6:**
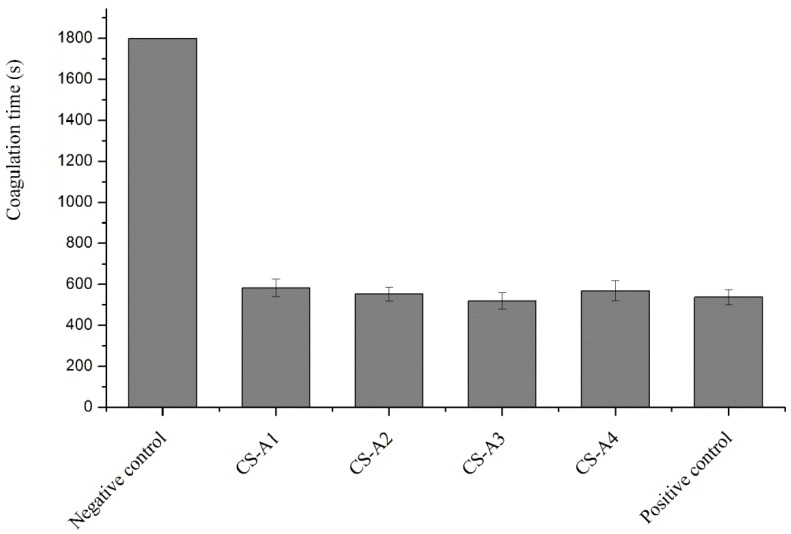
Effect of chitosan acid salts on coagulation time.

Chitosan is insoluble in water, soluble in acidic solutions, and many properties of chitosan exist only in its acid solutions. Chitosan dissolved in different acidic solutions might have different influences on its hemostatic activity. The effects of different chitosan acid salts on the coagulation time of rabbit blood are illustrated in Figure 6. Compared with the negative control group, all chitosan salts significantly promoted coagulation of fresh anticoagulant rabbit blood in vitro. Chitosan lactate (CS-A3) showed stronger procoagulant ability when compared with the positive control group. Studies showed that human whole blood rapidly formed a coagulum once treated with chitosan acid solutions [41]. The hemostatic properties of CS were possibly due to its interaction with RBCs resulting in agglutination of RBCs. That was partly affected by the protonation degree of the amino groups in CS chains, but also by its molecular structure.

From the comparison between CS-A2 and CS-A3, CS-A3 showed better performance in promoting coagulation, which was consistent with that reported in the literature [42]. This was probably because lactate, with a lower acidity coefficient (3.85) than that of acetic acid (4.75), had stronger ability to transfer protons and to form protonated amino groups.

### 2.7. Dynamic Coagulation Time

**Figure 7:**
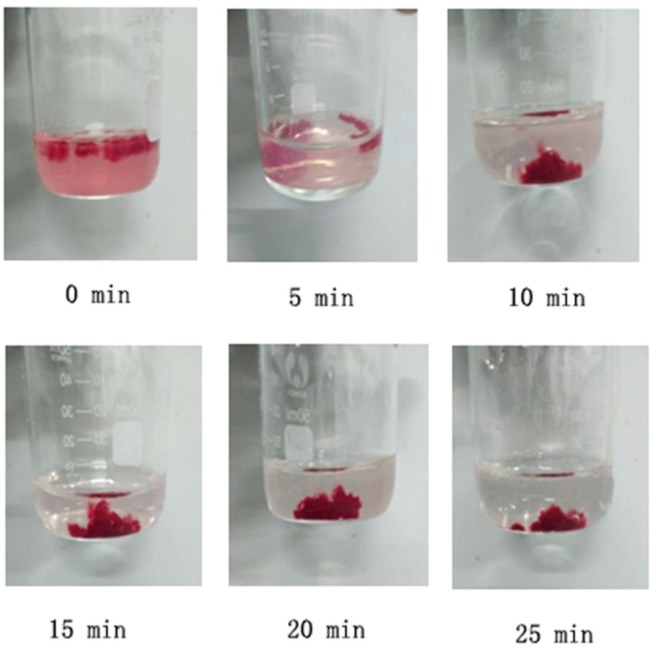
Dynamic coagulation process of chitosan (CS-D3).

**Figure 8:**
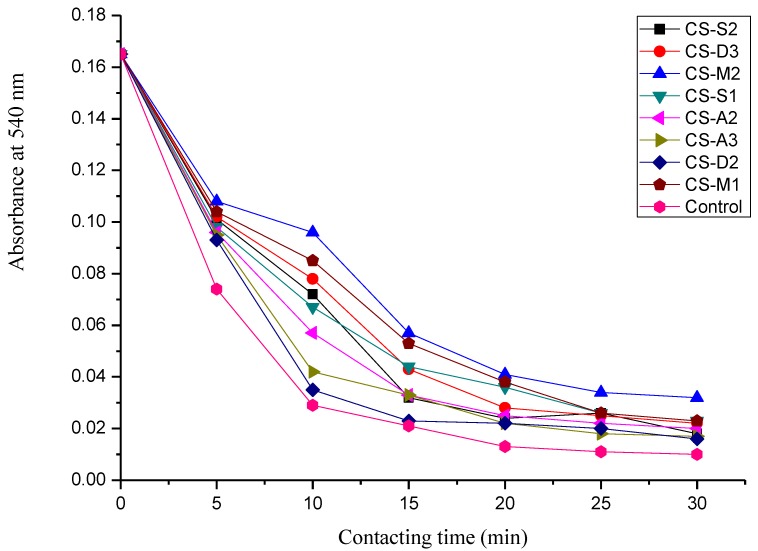
The dynamic coagulation curves of chitosan with various molecular parameters.

The dynamic coagulation process can reflect the response process of blood from the initial state to the final state after blood contacts with samples, as shown in Figure 7. Dynamic coagulation time is derived from the absorbance values of hemoglobin plotted versus the contacting time [43]. It is usually used to evaluate the effects of biomaterials on blood coagulation [44]. Figure 8 presents the dynamic coagulation time curves of chitosan with various molecular parameters. The steeper the curve and the shorter coagulation time, the stronger procoagulation ability of biomaterials. As shown in Figure 8, the positive control exhibited a sharp optical density value decrease within 10 min and chitosan with different molecular parameters presented varied dynamic coagulation behaviors. The dynamic coagulation curves of chitosan salts, particularly chitosan lactate (CS-A3), descended sharply, indicating excellent blood procoagulant performance. From the dynamic coagulation curves of chitosan with varied MW and DD, with approximate MW (CS-D2 and CS-D3) the procoagulant effect of chitosan improved with the decrease of DD. Additionally, with the same DD (CS-D3, CS-M1 and CS-M2), the greater the MW of chitosan was, the better the coagulation effect was. As for the substituent derivatives (CS-S1 and CS-S2), water-soluble substituent improved the blood coagulation effect of chitosan when compared to the original chitosan (CS-D3). These results were consistent with those of the above procoagulant activity assay in vitro.

### 2.8. Plasma Recalcification Time

Hemostasis is a very complicated cascade process which involves platelets, specialized blood cells, and a variety of molecules, leading to the formation of insoluble fibrin chains [45]. Hemostatic effects of the tests depend on many factors, such as thrombin and antithrombin, endogenous and exogenous coagulation factors, platelets, and so on. Thus, there are a number of indicators for in vitro investigation of hemostatic effects, including bleeding time, coagulation time, prothrombin time, prothrombin activity, plasma recalcification time, and platelet adhesion and aggregation rate. Generally, one or more indicators can be used to screen hemostatic agents. Plasma recalcification time is the time required for recovery of the intrinsic blood coagulation process after adding Ca^2+^ to the calcium removed plasma. The plasma calcium recovery test is a simple and direct method to simulate an intrinsic coagulation process in vitro. It is mainly used in the evaluation of the ability of platelets to form thrombus, which is important in hemostasis studies [46]. Therefore, the plasma recalcification time of chitosan was also investigated herein.

**Figure 9:**
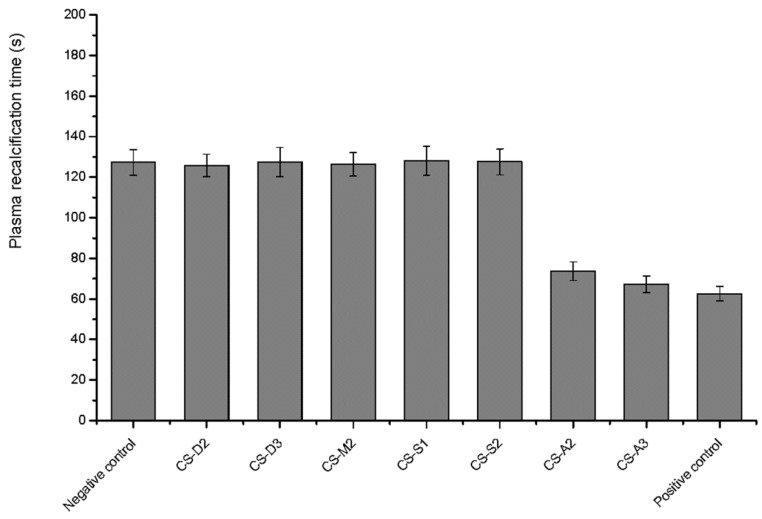
The plasma recalcification time of chitosan with various molecular parameters.

Figure 9 shows the results of the plasma calcium recovery test. The plasma recalcification time of the *Yunnan Baiyao* group (positive control) and the chitosan salt groups (CS-A2 and CS-A3) were significantly shorter than that of the negative control group (*p* < 0.01), which showed good intrinsic coagulant properties. The intrinsic coagulation factors (VIII, IX, XI, and XII) were involved in mammalian blood coagulation cascade [47]. However, it was not clear whether chitosan acid salts affected the above coagulation factors. In contrast, there was no significant difference between the chitosan-treated groups and the negative group (*p* > 0.05), suggesting that chitosan and chitosan salts may not have exactly the same blood procoagulant mechanisms. Some studies showed that chitosan facilitated hemostasis, possibly through interaction with erythrocytes, linking them together to establish a cellular clot or hemostatic plug [31,36]. In wounds, the activation of platelet adhesion and aggregation played a vital role in the process of hemostasis. Chou et al. demonstrated that chitosan enhanced the rabbit platelet adhesion and aggregation by significant increasing of platelet intracellular calcium mobilization and enhancing expression of glycoprotein IIb/IIIa complex on platelet membrane surfaces, which might account for its hemostatic effects [48]. Additionally, Suzuki et al. demonstrated that chitosan could cause complementary activation by acting directly to change C3 to C3i, or indirectly to activate serum proteases [49]. Although various possible hemostasis pathways have been reported, the hemostatic mechanism of chitosan has not been defined so far. Nevertheless, most of the studies indicated that the hemostatic mechanisms of CS were independent of the classical coagulation cascade.

## 3. Materials and Methods 

### 3.1. Materials

Chitin (1150 kDa MW and 10% DD) was purchased from Zhejiang Gold Shell Biochemical Limited Corporation, Wenzhou, China. Glacial acetic acid, phosphotungstic acid, and acetone were purchased from Sinopharm Chemical Reagent Company, Shanghai, China and were used as received. All the other chemicals were supplied by Kecheng Trading Co., Ltd., Zhanjiang, China and were used without further purification.

### 3.2. Preparation of Chitosan with Different Molecular Parameters

#### 3.2.1. Preparation of Chitosan with Different Degrees of Deacetylation

Chitin powder (5% *w*/*v*) was added into 45% sodium hydroxide solution, heated and stirred at 110 °C under a nitrogen atmosphere for 10 h, 15 h, 20 h, and 25 h, respectively. The mixture was then filtered. The residue was washed with purified water until the washing water was neutral and then dried to obtain chitosan. The DD of chitosan was calculated with the element analysis method [50].

#### 3.2.2. Preparation of Chitosan with Different Molecular Weights

Chitosan was degraded with hydrogen peroxide under the catalysis of phosphotungstic acid [51]. Briefly, chitosan (7.5% *w*/*v*), phosphotungstic acid (0.1% *w*/*v*), and 30% hydrogen peroxide (15% *v*/*v*) were added into purified water. The mixture was left to react at 70 °C for 10, 15, 20, and 25 min, respectively, and then filtered. The residue was washed with deionized water until the washing solution was neutral and then vacuum dried. The viscosity average molecular weight of chitosan was calculated by measuring its intrinsic viscosity [35]. Briefly, chitosan was dissolved in CH_3_COONa (0.1 mol/L)-CH_3_COOH (0.2 mol/L) solution, and then an Ubbelohde viscometer (Shanghai Shenyi Glass Products Co., Ltd., Shanghai, China) was used to determine the intrinsic viscosity at 30 °C by the gradient dilution method. A regression curve could be made by plotting the concentration of chitosan solution (C) as the x-axis and the ratio of specific viscosity to C (η_sp_/C) as the y-axis, then the linear extrapolation method was used to make C equal to zero, and the *y* axis intercept as the intrinsic viscosity ([η]) was obtained. The viscosity average molecular weight of chitosan (*M_v_*) was calculated by the Mark–Houwink equation as follows.

(1) $$\left[\eta \right]=KM_{v}^{\alpha },$$

where *K* = 1.64 × 10^−30^ × DD^14.0^ (r = 0.996), and *α* = −1.02 × 10^−2^ × DD + 1.82 (r = 0.998).

#### 3.2.3. Preparation of Chitosan by Hydrophilic Modification

*O*-hydroxypropyl chitosan and *O*-carboxymethyl chitosan were prepared from chitosan with reference to methods described in the literature [52,27], respectively.

#### 3.2.4. Preparation of Chitosan Acid Salts

Both chitosan acetate and gentisate were prepared at room temperature by immersing chitosan into 4 mol/L mixed solutions of acids and isopropanol (1:3 *v*/*v*) and the reaction mixture was stirred at room temperature for 3 h. Chitosan iodate and lactate were prepared at a low temperature by immersing chitosan into 6 mol/L acids and the reaction mixture was stirred at 4 °C for 24 h. After that, the complexes were washed with 75% and 100%isopropanol, respectively, and then dried in vacuum.

### 3.3. Fourier Transform Infrared (FTIR) Spectroscopy

FTIR spectroscopy was used to investigate the chemical structure of chitosan. The KBr-supported discs of chitosan samples were measured by using an infrared spectroscopy (Perkin Elmer Spectrum 100, Waltham, MA, USA) in the frequency range of 4000–450 cm^−1^ at a resolution of 4 cm^−1^.

### 3.4. Procoagulant Activity Assay in Vitro

New Zealand rabbit blood was collected with evacuated blood tubes (sodium citrate as an anticoagulant to blood ratio 1:9, *v*/*v*) and kept at 4 °C for use. The procoagulant activity assay in vitro was carried out by using the test tube method [53] with slight modification. Briefly, 50 mg of chitosan powders were added to two tubes as the experimental groups, taking *Yunnan Baiyao* powder as the positive control and a blank tube as the negative control. The tubes were gently shaken to spread the powders to the bottom of tubes and then placed in a 37 °C water bath. The freshly drawn anticoagulated blood was quickly taken into two glass tubes (1 mL per tube), and one tube was slowly tilted once every 30 s, whereas the second tube was kept undisturbed. When a blood clot appeared in the first tube, the second tube began to tilt every 30 s. Timing stopped when the blood in the second tube began to coagulate. The time from the start of blood addition to the blood coagulation in the second tube was recorded as coagulation time.

### 3.5. Dynamic Blood Coagulation Assay

A proper amount of water-insoluble chitosan was dissolved in 1% acetic acid aqueous solution and a proper amount of water-soluble chitosan was dissolved in deionized water to prepare 10 mg/mL sample solutions. The above sample solutions (1 mL) were added to the watch glass and placed in a vacuum drying oven to form films through vacuum drying. Films prepared with water-insoluble chitosan were soaked in 0.1 mol·L^−1^ sodium hydroxide solution for 10 min, washed with distilled water until the washing solution was neutral, and then vacuum dried again. To each watch glass, 0.5 mL of fresh anticoagulant rabbit blood and 50 uL of calcium chloride solution (0.25 mol·L^−1^) were added while the stopwatch started at the same time. Every 5 min, 100 mL of distilled water was slowly poured into the watch glass. Injection fluid was collected and centrifuged. The supernatant was measured for absorbance at 540 nm. The surface of the watch glass was directly used as the positive control. The dynamic coagulation curve of blood contact time versus absorbance was drawn.

### 3.6. Determination of Plasma Recalcification Time

The plasma recalcification time was determined according to the method described in the literature [54] with a few modifications. Briefly, the New Zealand rabbit blood was collected with an evacuated blood tube containing sodium citrate as an anticoagulant (volume ratio of anticoagulant to blood: 1:9) and centrifuged at 3000 rpm for 15 min (Certificate No. SYXK20150147). Afterwards, the supernatant was separated to obtain platelet-poor plasma (PPP). To each Eppendorf tube, 100 µL of PPP was added and then 100 µg of chitosan powder was added as the experimental group. *Yunnan Baiyao* powder and a blank tube were used as the positive and negative control group, respectively. All the tubes were incubated in a 37 °C water bath for 3 min and then 0.1 mL of 0.025 mol/L calcium chloride solution was added, well mixed, and placed in a 37 °C water bath again while the stopwatch was started until white jelly began to occur in the mixed solution.

### 3.7. Statistical Analysis

All the experimental values were expressed as means ± standard deviation ($\bar{x}$ ± SD). The comparison analysis between the groups was carried out using the analysis of variance (ANOVA) with the Statistical Package for Social Sciences software (SPSS 21.0, International Business Machines Corporation, Armonk, NY, USA), and *p*-value of less than 0.05 was considered to be statistically significant.

## 4. Conclusions

Some chitosan with multiple polymer parameters including degree of deacetylation, molecular weight, substituent group, and acid salt was successfully prepared from chitin. The procoagulant evaluation in vitro and dynamic blood clotting assay demonstrated that the molecular parameters investigated all produced remarkable effects on the hemostatic properties of chitosan. In particular, chitosan with a moderate degree of deacetylation and chitosan lactate had strong procoagulant activities. Nevertheless, the relationship between the structure of chitosan and the procoagulant properties still remains a great challenge. The results of plasma recalcification time showed that chitosan with different molecular parameters may not have exactly the same blood procoagulant mechanisms. Although chitosan can stop bleeding through a variety of approaches, its hemostatic mechanisms are still inconclusive. Therefore, a lot of effort is still needed to develop chitosan as a natural and effective hemostatic material in clinical applications.

## Figures and Tables

**Table 1:** Chitosan with different molecular parameters. DD: degree of deacetylation; MW: molecular weight; DS: degree of substitution; SGs: substituent groups; CS: chitosan.

Chitosan with Different Molecular Parameters		DD (%)	MW (kDa)	DS (%)	Marked
Chitosan with different DD		52.68	1010	-	CS-D1
		68.36	933	-	CS-D2
		81.72	891	-	CS-D3
		92.21	877	-	CS-D4
Chitosan with different MW		81.72	485	-	CS-M1
		81.72	212	-	CS-M2
		81.72	56	-	CS-M3
		81.72	27	-	CS-M4
Chitosan with different SGs	*N*-Acetyl	10	1150	90	Chitin
	*O*-carboxymethyl	-	-	58.36	CS-S1
	*O*-hydroxypropyl	-	-	63.13	CS-S2
Chitosan-acid salts	Chitosan-HI	-	-	-	CS-A1
	Chitosan-CH_3_COOH	-	-	-	CS-A2
	Chitosan-lactic acid	-	-	-	CS-A3
	Chitosan-gentisic acid	-	-	-	CS-A4
